# Prevalence and Clinical Characterization of Bocavirus Infection in a Specialized Children's Hospital in Saudi Arabia

**DOI:** 10.7759/cureus.22127

**Published:** 2022-02-11

**Authors:** Hamad Alkhalf, Ashwag R Almutairi, Abeer Almutairi, Reem K Almutairi, Suliman AlGhnam, Sameera Aljohani, Jubran T Alqanatish, Amir Babiker

**Affiliations:** 1 General Pediatric, King Saud Bin Abdulaziz University for Health Sciences College of Medicine, Riyadh, SAU; 2 Pediatrics, King Abdulaziz Medical City Riyadh, Riyadh, SAU; 3 Pediatrics, King Abdullah International Medical Research Center, Riyadh, SAU; 4 Pediatrics, King Saud University, Riyadh, SAU; 5 Pediatrics, King Saud Bin Abdulaziz University for Health Sciences College of Medicine, Riyadh, SAU; 6 Nursing, King Saud Bin Abdulaziz University for Health Sciences, Riyadh, SAU; 7 Population Health, King Abdullah International Medical Research Center, Riyadh, SAU; 8 Infectious Diseases, King Saud Bin Abdulaziz University for Health Sciences College of Medicine, Riyadh, SAU; 9 Infectious Diseases, King Abdulaziz Medical City Riyadh, Riyadh, SAU; 10 Infectious Diseases, King Abdullah International Medical Research Center, Riyadh, SAU; 11 Pediatrics, King Abdulaziz Medical City and King Saud Bin Abdulaziz University for Health Sciences, National Guard Health Affairs, Riyadh, SAU; 12 Pediatric, King Saud Bin Abdulaziz University for Health Sciences College of Medicine, Riyadh, SAU

**Keywords:** bocavirus, saudi arabia, kasch, respiratory, length of stay

## Abstract

Aim

The aim of this study was to assess the prevalence and clinical characterization of bocavirus infection in patients admitted with respiratory symptoms to a specialized children’s hospital in Riyadh, Saudi Arabia.

Methods

This is a retrospective cross-sectional study that included children aged 0-14 years and was conducted over a two-year period (2017-2019). All data were gathered from an electronic information recording system, which included patients’ demographics, comorbidities, clinical presentation, complication, and duration of hospitalization.

Results

Among all patients (11,709) admitted to King Abdullah Specialized Children’s Hospital with predominant respiratory symptoms during the study period, 193 (1.6%) patients had bocavirus infections. Most of the patients were diagnosed in winter months. Cough was the primary presenting symptom (91.7%) followed by fever (83.4%). Gastrointestinal symptoms were also common (anorexia in 62% and vomiting in 39%). In 80% (n=154/193) of cases, bocavirus co-existed with other viruses, namely, human rhinovirus (45.8%), human adenovirus (31.2%), and respiratory syncytial virus type A (17.5%). Moreover, those who required oxygen supply stayed longer in the hospital (p<0.001) and were more likely to receive multiple medications such as bronchodilators (p<0.001), corticosteroids (p<0.001), and nebulized racemic epinephrine (p>0.05). Children infected with bocavirus and co-existing viruses were less likely to require oxygen supply (p<0.050).

Conclusion

Bocavirus infection is more common during winter months and predominantly affects respiratory and gastrointestinal systems in children. More studies are needed to evaluate the global impact of this recently recognized infection.

## Introduction

Bocavirus is a recently described virus that mainly affects lower respiratory and gastrointestinal tracts of children [1]. Bocavirus is a type of parvovirus family and is known to have a small size of 20 nm [1]. The name “bocavirus” is derived from a combination of bovine parvovirus and canine minute virus, which have similar genetic and amino acid structures [2]. Human bocavirus (HBoV) is non-enveloped with a single-stranded negative DNA virus [2]. Moreover, four strains of this virus have recently been identified: HBoV1, HBoV2, HBoV3, and HBoV4 [3]. HBoV1 has mainly been identified in respiratory specimens. Other types of bocavirus have been identified in stool specimens [3]. Moreover, bocavirus was also detected in urine, saliva, and blood, and had been identified in sewage and river water [2].

Bocavirus usually affects infants and children from six months to two years of age, although some cases have been found in children older than five years of age [1]. It can be detected alone or, more commonly, in conjunction with other viruses that cause respiratory or gastrointestinal infections such as human rhinovirus, adenovirus, norovirus, and rotavirus [4]. In particular, co-infection with respiratory syncytial virus (RSV) has been found in 89% of cases [5]. Therefore, it was thought that HBoV might be a harmless passenger rather than a true pathogen [5,6]. Bocavirus presents with non-specific symptoms such as cough, wheeze, pneumonia, and fever [3]. Also, especially with HBoV2 and HBoV3, it can present with acute gastroenteritis [3].

Rhinorrhea, asthma exacerbation, and bronchiolitis were detected in some young patients in conjunction with a progressive bocavirus infection [7]. Two of the rare and life-threatening conditions that bocavirus-infected children can develop are pneumomediastinum and bilateral pneumothorax [7]. HBoV is exclusively detected by molecular detection methods [8]. Currently, laboratories use either in-house polymerase chain reaction (PCR) or real-time PCR assays in order to detect the virus [8]. Moreover, the PCR method targets NP-1, NS-1, or VP1/2 genes, which are proteins or genes found on this virus [8,9]. Recently, some multiplexing assays have been developed, which are the RespiFinder assay (PathoFinder, Maastricht, the Netherlands) and the Luminex xTAG Respiratory Viral Panel (RVP) FAST v2 assay (Luminex Molecular Diagnostics, Toronto, ON, Canada) [1,9]. The latter has been approved by the U.S. Food and Drug Administration (FDA) [1,9]. So far, there is no antiviral medication known to treat HBoV [10]. Similar to many other viruses, supportive therapy to control symptoms remains the mainstay of treatment for HBoV [10]. This includes providing oxygen supply for hypoxia and the use of bronchodilators for patients with wheezes [1,10].

The first isolation of this virus was found in 2005 in upper respiratory secretions in acutely ill patients in Sweden [4]. The estimated worldwide rate of HBoV is 2-20%, but the true comparable rate cannot be detected due to lack of studies in different populations [1]. Locally, one study conducted in Al-Taif, a city in the western region of Saudi Arabia, determined the prevalence of bocavirus in 80 samples of nasopharyngeal swabs [11]. In this study, bocavirus had been detected in 22% (18/80), mainly in young children between five months and two years of age [11]. Our study aimed to examine the prevalence and clinical characterization of bocavirus infection among children in Saudi Arabia to raise awareness about the disease among the health personnel in public health care authorities.

## Materials and methods

This is a retrospective cross-sectional study at King Abdullah Specialized Children’s Hospital (KASCH) in Riyadh, Saudi Arabia. We included all patients aged 0-14 years admitted as inpatients in KASCH with bocavirus infection who had respiratory, gastrointestinal, or other symptoms from November 2017 until November 2019. Data included all patients who tested positive for bocavirus, isolated or with co-infection. We excluded admissions in the emergency room and daycare unit during the study period. The estimated sample size was 660 patients using the online Raosoft sample size calculator. This was based on a precision of 10 percentage points of the true prevalence, with a confidence level of 95% and an expected prevalence of 22% [11]. However, we included all patients who met the inclusion criteria. The data were collected from an electronic health record system known as BestCare. The data collection sheet was designed to capture different variables including clinical and laboratory findings. Information about the level of baseline clinical comorbidities, duration of oxygen therapy, length of hospital stay (LOS), complications, and, lastly, mortality (if any) was also planned to be reported. The study was reviewed and approved by the Institutional Review Board of King Abdullah International Medical Research Center (KAIMRC).

Statistical analysis

The data were analyzed using SPSS (IBM Corp., Armonk, NY, USA). Data were presented as mean ± standard deviation (SD) for continuous variables and as percentages for categorical variables. The chi-square test or Fisher’s exact test was used to compare categorical data. A logistic regression was constructed to determine independent significant predictors of an extended hospitalization, defined as an LOS above the mean of 5.3 days. Odds ratios (OR) with 95% confidence intervals (CI) were expressed relative to a reference baseline category. A p-value of 0.05 was used as a cut-off for statistical significance.

## Results

Among all patients (11,709) admitted to King Abdullah Specialist Children Hospital with respiratory illness during the study period, we included 193 (1.6%) patients who had bocavirus infections (Table 1). The frequency of reported symptoms and signs varied between patients with a mean of 6 ± 2 complaints and clinical findings per admitted child with bocavirus infection (Table 2). Patients were treated using variable medications according to their clinical presentation (Table 2). Analysis showed that there were at least 224 co-viral infections in 154 (80%) of children admitted with bocavirus infection, with the highest association with human rhinovirus (45%) and human adenovirus (30%). Other viruses with less co-infection association with bocavirus in our cohort include RSV (7%) and less than 5% for each of the following viruses: influenza and parainfluenza viruses, human coronavirus OC43 and NL63, Epstein Barr virus, and rotavirus. Of these patients with co-infections, 100 had one co-infection, 41 had two co-infections, 10 had three co-infections, and three patients had four co-infections.

**Table 1:** Characteristics of study subjects (N=193) BMI, body mass index; LOS, length of hospital stay; SD, standard deviation

	Frequency	Percentage
Sex		
Male	102	52.8
Female	91	47.2
Age (months), mean (SD)	23.04 (22.5)	
Infant (0-11.9 months)	68	38.6
Toddler (1-3 years)	75	42.6
Pre-school aged (3.1-5 years)	23	13.1
School aged (5.1-12 years)	8	4.5
Adolescent (12.1-14 years)	2	1.1
Weight (kg), mean (SD)	10.68 (5.4)	
Height (cm), mean (SD)	80.27 (16.6)	
Body mass index, mean (SD)	15.73 (2.68)	
Age-adjusted BMI classification		
Underweight for age ≤ 5th percentile	43	22.3
Normal weight (>5th-90th percentile)	117	60.6
Over-weight >90% percentile	33	17.1
Length of hospital stay (days), mean (SD)		
LOS≤5.3 days	147	76.2
LOS>5.3 days	46	23.8
Comorbidity		
No	118	61.1
Yes	75	38.9
Year of admission		
2017	17	8.8
2018	111	57.5
2019	65	33.7
Admission quarter of the year		
1st quarter	78	40.4
2nd quarter	44	22.8
3rd quarter	25	13
4th quarter	46	23.8

**Table 2:** Clinical characterization of bocavirus -infected children, symptoms and signs, and received medical treatment NSAIDs, non-steroidal anti-inflammatory drugs; SD, standard deviation

	Frequency	Percentage
Presenting respiratory manifestations		
Cough	177	91.7
Shortness of breath	140	72.5
Nasal discharge	87	45.5
Wheezing	81	42
Congested throat	80	41.5
Chest retractions using accessory muscles	58	30.1
Stridor	7	3.6
Cyanosis	1	0.5
Presenting gastrointestinal manifestations		
Loss of appetite	124	64
Vomiting	76	39.4
Diarrhea	25	13
Abdominal pain	1	0.5
Splenomegaly	1	0.5
Presenting immune system manifestations		
Fever	161	83.4
Fatigue	112	58
Tonsillitis	8	4.1
Lymph node enlargement	6	3.1
Convulsions	5	2.6
Other body systems’ presenting manifestations		
Ear congestion	12	6.2
Conjunctivitis	10	5.2
Skin rash	9	4.7
Eye discharge	3	1.6
Ear discharge	2	1
Photophobia	1	0.5
Total symptoms and signs (severity), mean (SD)	6.15 (1.84)	
Medical management		
Bronchodilators	148	77.5
O_2_ supplement (0.2-1.5 L/minute)	113	59.2
Antibiotics	111	58.1
Antipyretics	96	50.3
Corticosteroids	91	47.6
Pain killers (NSAIDs)	36	18.8
Other medications	28	14.7
Racemic epinephrine (nebulized)	22	11.5
Duration (hours) of supplemental O_2_, mean (SD)	10.74 (6.4)	
Other interventions		
Blood cultures	1	0.5
Urine culture	4	2.1
Respiratory culture	2	1

Laboratory results indicate that the mean leukocytes count for the patients upon admission was normal (4.0 - 11.0 x10^9^/L) in 73.6% of cases compared to 8.8% of children admitted with lower than (4.0 x10^9^/L) white blood cell count. Moreover, the mean hemoglobin level (gm/L) was normal for age in 82.4% of cases. Additionally, serum glucose levels were higher than the normal range for age in the majority (89.6%) of children, but only 10.4% of them had normal blood glucose and none of them had low serum glucose level. The majority of children with bocavirus infection (76.2%) showed signs of hyponatremia with serum Na < 135 mmol/L. A smaller number of them had a normal serum Na+ level (135-145 mmol/L), and none of these patients had hypernatremia. Moreover, most of the children (64.8%) had a normal serum potassium level (K+) upon admission and 27.5% presented with hypokalemia. Few (7.8%) of these patients had hyperkalemia.

**Figure 1:**
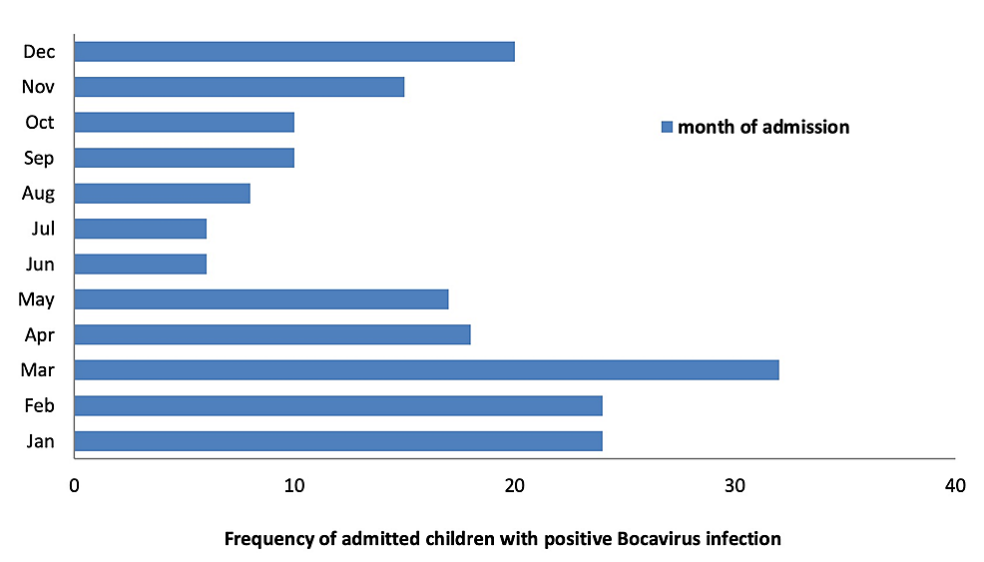
Frequency of monthly admitted positive bocavirus-infected children with respiratory complaints

An overall average LOS for patients was 5.31 + 7 days, with 75% of children stayed for five or less days in the hospital. Based on the mean score (LOS=5.3), we divided patients based on their LOS into two groups of short LOS (≤5.31 days) and extended LOS (>5.3 days). Furthermore, we described the quarterly admission of the children with respiratory complaints who were diagnosed with a positive bocavirus infection in order to identify any seasonal variations in the numbers of admissions per year, month, and quarter (Figure 1 and Table 1). Most of these cases appeared to occur during the first three months (i.e., the winter quarter) of the year, and fewer cases were seen during the summer quarter (June-August). The number climbed again during the fall quarter (i.e., between September and December) (Figure 1). The most common reported complication with bocavirus infection in our cohort was respiratory distress (Table 3).

**Table 3:** Measured complications of hospitalized children with bocavirus infection (N=79)

Complication	Frequency	Percentage
Respiratory distress	75	94.9
Dehydration	3	3.6
Respiratory failure requiring ventilatory support	2	2.5
Pneumothorax	1	1.3
Septic shock	1	1.3
Superimposed lymphadenitis	1	1.3

Regression analyses identified comorbidities and the need for oxygenation during management as the significant predictors of extended LOS (> 5.3 days) (Table 4). After adjusting for age, gender, and body mass index (BMI), having at least one comorbidity was associated with a fourfold increased odd of extended stay (OR=4.5; 95% CI=2.1-9.5). Irrespective of age, gender, or BMI, patients who received oxygen therapy required as twice as the duration of hospital stay of those without oxygen requirement (OR=2.2; 95% CI=1.0-9.5). 

**Table 4:** Logistic regression model of the association between patients’ characteristics and extended hospital stay (defined as > 5.3 days) *Significant at 0.05 level BMI, body mass index; CI, confidence interval; LOS, length of hospital stay; OR, odd ratio

Variable	Extended LOS unadjusted, OR (CI)	Extended LOS adjusted, OR (CI)
Age at admission time (months)	1.0 (0.99-1.0)	1.0 (0.98-1.0)
Gender	1.0 (0.53-2.0)	0.91 (0.44-1.8)
BMI	0.84 (0.73-.97)	0.88 (0.76-1.0)
O_2_ treatment (yes vs. no as reference)	2.4 (1.1-5.0)	2.2* (1.0-4.9)
Comorbidity (yes vs. no as reference)	5.5 (2.6-11.3)	4.5* (2.1-9.5)

## Discussion

Bocavirus infection was less common in our study group compared to worldwide reports and previous prevalence studies in Saudi Arabia [11-16]. Affected children were of younger age group and were more prone to get infected during winter months. Respiratory symptoms were non-specific, but there were also gastrointestinal symptoms in most of our patients. In our tertiary institute, the majority of patients received oxygen, antibiotics, corticosteroids, and bronchodilators, but the LOS was relatively short in duration compared to other viruses.

HBoV has variable prevalence in different parts of the world [12-16]. Similar to the findings in previous international studies, our study showed low prevalence of bocavirus infection among inpatient children [9,17,18]. Nevertheless, in a previous study in Al Taif in Saudi Arabia, which lies in a high altitude (1.879 meters, 6.16 feet in the slopes of the Hejaz mountains), a notably higher prevalence of bocavirus infection of 22.5% was reported [11]. This could be related to more cool weather during winter season that enhances viral replication, which is the same cause of higher reports of the disease during winter months in our cohort [11]. On average, temperature in Riyadh ranges from 49°F to 110°F (rarely below 41°F or above 114°F). The age of children who were infected with bocavirus was lower in our study compared to a previous study in Al Qatif, Saudi Arabia [19]. However, our results were in keeping with the age distribution reported in most of the other studies with predominance in children less than 24 months old possibly related to suboptimal immune response against the virus in this age group [4,9,20]. Unlike most of previous pediatric studies on bocavirus infection that reported male predominance, our study showed almost equal gender distribution [11,19,21]. Understandably, younger age group in our cohort, likewise in other studies, had more respiratory complaints compared to older age group. However, systemic symptoms and immunological manifestations such as fever, lymph node inflammation, body aches, and convulsions accompanying fever. Similar to many other viruses, bocavirus had the highest incidence in our study during winter and fall quarters of the year [4,17,18]. On the other hand, some studies found bocavirus to be detected more in the summer season [11,22]. Another study in China found that the incidence of bocavirus increased more during the spring and summer seasons (May-June 2010) [23].

Characterizing viral respiratory tract infections is difficult because various symptoms can be caused by a wide range of viruses. In all studies of bocavirus, including ours, researchers have agreed that bocavirus usually presents predominantly as a lower respiratory tract infection [21,23,24]. In consistence with other bocavirus studies, our study showed a significant rate of co-infection with other viruses [25-28]. The most common coexisting viruses in our study were human rhinovirus, human adenovirus, and RSV. This phenomenon could be explained by the fact that bocavirus weakened children’s immunity, and, therefore, children were more likely to get other infections, or it could be that the pathogenesis of bocavirus led to introduction of other viruses to the systems. Another possibility is that bocavirus is acting as a bridge for other viruses or it needed other virus(es) to replicate. Moreover, our study indicated that bocavirus could present with gastrointestinal symptoms such as vomiting and diarrhea, which have also been reported in previous studies [9,24,29]. However, the most common reason for hospital admission with bocavirus infection in children is respiratory distress. We found that 75/193 children we studied experienced respiratory distress of various degrees of severity. This was also observed in a study in Thailand, which showed that the bocavirus could cause severe respiratory distress that led to pneumonia [25]. An explanation of this could be due to the pathogenesis of bocavirus or because of the higher rate of co-infections. It could also be related to immaturity of the immune system in children younger than two years of age contributing to the severe forms of the infection [25,26]. In our study, bocavirus-infected children with predominantly gastrointestinal complaints and/or those with coexisting positive viral infection tended to have more of an immune response resulting in apparent constitutional and systemic manifestations such as fever, tiredness, and lymphadenopathy.

Most patients had shorter LOS in our study. Another study in China also found that bocavirus-positive admitted patients had a short period of hospitalization compared to other common viruses [22]. The main predictors of extended LOS in our study were comorbidity and the requirement of oxygen treatment. A similar finding with regard to oxygenation being an influencing factor of LOS of children with bocavirus infection was reported in a previous study in California [30]. Interestingly, as also observed in a study in Brazil, children who received oxygen in our study were younger (infants and toddlers) and stay longer in the hospital with the bocavirus infection. When adjusted in our logistic regression analyses, the prolonged LOS was mainly related to the oxygen requirement but not to the age factor. Also, as expected, comorbid patients had a greater need for oxygen therapy in our study than those who are otherwise healthy, and that certainly adds to the prolonged LOS. Last but not least, at all times and especially in this era of post-COVID-19 pandemic, education about hand washing and other measures of infection control for parents and caregivers of hospitalized children should not be forgotten as this would certainly improve LOS.

Most patients received bronchodilators, antipyretics, antibiotics, and steroids in our study, irrespective of complexity level of their medical condition. The top administered medical therapy in our study was bronchodilators followed by oxygen therapy. The mean oxygenation duration was 10.74 + 6.4 hours on average. Children infected with bocavirus and co-existing viruses were less likely to require oxygen supply. Although RSV is usually associated with oxygen requirement, it only coexisted with bocavirus infection in 7% of our cohort. Given this interesting observation the study lent on the oxygen requirement, the authors postulate that coexistence with other viruses probably resulted in a better immune response observed as more apparent constitutional symptoms in these patients. This could also hypothetically reduce the severity of bocavirus infection and related oxygen requirement in these patients. Steroids, specifically prednisolone, were not found effective in treating children with bocavirus [10]. Around 47% of our patients, however, were treated with steroids, and around 60% of those who received oxygen were also treated with steroids. Most of the children in our study were placed on at least one kind of antibiotic therapy during hospital admission despite the viral nature of the disease. An additional diagnosis of a possible bacterial infection such as otitis media or pneumonia in these patients has warranted the use of the antibiotic. However, the use of antibiotics was empirical in most of these patients based on the severity of presentation and suspicion of co-existing bacterial infection. Therefore, a stewardship antimicrobial program was implemented in our institute to minimize the overuse of antibiotics in such patients. Understandably, the majority of bocavirus admitted cases received anti-pyretic medications to relieve their fever.

A limitation of our study was that it was a single-center study conducted over a limited period of time. Probably, a larger multicenter study is required to evaluate the global impact of this virus over a longer period of time.

## Conclusions

Bocavirus is a recently recognized virus that predominantly affects respiratory and gastrointestinal systems. Similar to other viruses, it tends to affect children more during winter months with a complicated course in vulnerable patients who have other comorbidities; hence, patients had a prolonged LOS in hospital. Oxygen and bronchodilators were commonly used modalities of management, and steroids were not found to be effective in our cohort.
